# Volume Regulated Anion Channel Currents of Rat Hippocampal Neurons and Their Contribution to Oxygen-and-Glucose Deprivation Induced Neuronal Death

**DOI:** 10.1371/journal.pone.0016803

**Published:** 2011-02-11

**Authors:** Huaqiu Zhang, H. James Cao, Harold K. Kimelberg, Min Zhou

**Affiliations:** 1 Ordway Research Institute, Albany, New York, United States of America; 2 Department of Neurosurgery, Tongji Hospital, Tongji Medical College, Huazhong University of Science and Technology, Wuhan, People‚s Republic of China; University of Queensland, Australia

## Abstract

Volume-regulated anion channels (VRAC) are widely expressed chloride channels that are critical for the cell volume regulation. In the mammalian central nervous system, the physiological expression of neuronal VRAC and its role in cerebral ischemia are issues largely unknown. We show that hypoosmotic medium induce an outwardly rectifying chloride conductance in CA1 pyramidal neurons in rat hippocampal slices. The induced chloride conductance was sensitive to some of the VRAC inhibitors, namely, IAA-94 (300 µM) and NPPB (100 µM), but not to tamoxifen (10 µM). Using oxygen-and-glucose deprivation (OGD) to simulate ischemic conditions in slices, VRAC activation appeared after OGD induced anoxic depolarization (AD) that showed a progressive increase in current amplitude over the period of post-OGD reperfusion. The OGD induced VRAC currents were significantly inhibited by inhibitors for glutamate AMPA (30 µM NBQX) and NMDA (40 µM AP-5) receptors in the OGD solution, supporting the view that induction of AD requires an excessive Na^+^-loading via these receptors that in turn to activate neuronal VRAC. In the presence of NPPB and DCPIB in the post-OGD reperfusion solution, the OGD induced CA1 pyramidal neuron death, as measured by TO-PRO-3-I staining, was significantly reduced, although DCPIB did not appear to be an effective neuronal VRAC blocker. Altogether, we show that rat hippocampal pyramidal neurons express functional VRAC, and ischemic conditions can initial neuronal VRAC activation that may contribute to ischemic neuronal damage.

## Introduction

Volume-regulated anion channels (VRAC), also called volume-sensitive outwardly rectifying (VSOR) Cl^-^ channels or volume-sensitive organic anion channels (VSOAC), are a group of widely expressed Cl^-^ channels. The important roles of VRAC include regulating cell volume under physiological and pathological conditions through the mechanisms termed regulatory volume decrease (RVD) [1], [2], [3], [4], [5]. Although several chloride channels have been proposed as the underlying channels, the molecular identity of VRAC is yet unknown [6], [7], [8]. Therefore, identification of VRAC has still been based on the criteria of induction of an anion conductance in hypoosmotic medium, outward current rectification of whole-cell currents and sensitive of induced anion currents to a number of anion channel inhibitors.

In the mammalian central nervous system (CNS), VRAC have been mostly studied in primary cultured astrocytes in relation to their role in the pathological release of excitatory amino acids [1], [2], [9]. In a brain slice study, some cortical neurons showed a steadfast cell volume change to the osmotic stress [10]. However, VRAC, both in slices and primary cultures, could be activated from barrel cortex neurons in hypoosmotic medium, and Na^+^-overloading via glutamate NMDA/AMPA receptors has been shown to initiate neuronal VRAC activation [11], [12].

In the ischemic brain, disruption of energy supply can modulate cell swelling and VRAC activity and excessive ionotropic glutamate receptor activation is an early pathological event [13], [14], however, whether pathological stimulation of neuronal glutamate receptors could also underlie neuronal VRAC activation under cerebral ischemic conditions is unknown. A moderate activation of VRAC enables restoration of cell volume in the face of osmotic stress, but over activation of VRAC can lead to apoptotic or necrotic neuronal death depending on the severity of the conditions [5], [15], [16]. Therefore, whether activation of VRAC helps survival or imposes further damage to neurons in the stroke brain needs to be determined.

In the present study, we investigated the activation of VRAC of rat hippocampal pyramidal neurons in acutely prepared slices induced by hypoosmotic medium and oxygen-and-glucose deprivation (OGD) solution. We show that OGD-induced neuronal VRAC activation is largely a consequence of pathological stimulation of ionotropic glutamate receptor and contributes to the ischemia-induced neuronal death.

## Materials and Methods

### Hippocampal slice preparation

Hippocampal slices were prepared from 3–4 week old male Sprague-Dawley rats [17], [18] in accordance with a protocol (#03-379) approved by the Wadsworth Center, New York State Department of Health Institutional Animal Care and Use Committee. Animals were anesthetized with 20% CO_2_ (balanced with atmospheric air) before decapitation, and their brains were removed from the skull and placed in an ice-cold, oxygenated (5% CO_2_-95%O_2_, pH  = 7.35) slice preparation solution containing (in mM) 26 NaHCO_3_, 1.25 NaH_2_PO_4_, 2.5 KCl, 10 MgCl_2_, 10 glucose, 0.5 CaCl_2_, and 240 sucrose. Final osmolarity was 350±2 mOsm; a higher osmolarity used in preparation solution ensures the viability of neurons and astrocytes in slices [17], [18], [19]. Coronal slices of 300 µm thickness were cut with a Vibratome 1500 (Ted Pella Inc., Redding, CA, USA) and transferred to a nylon-basket slice holder in 20–22°C artificial cerebral spinal fluid (aCSF) containing (in mM) 125 NaCl, 25 NaHCO_3_, 10 glucose, 3.5 KCl, 1.25 NaH_2_PO_4_, 2.0 CaCl_2_, and 1.0 MgCl_2_ (osmolarity, 295±5 mOsm). The slices were allowed to recover in aCSF with continuous oxygenation for at least 60 min before recording.

### Isoosmotic, hypoosmotic and oxygen-and–glucose deprivation (OGD) conditions

We used the solutions reported by Inoue et al., [11] to selectively measure VRAC from neurons in brain slices. Briefly, the neuronal Na^+^ channel currents were inhibited by tetrodotoxin (TTX) in the bath solution and the K^+^ conductance was inhibited by substituting choline or NMDG for K^+^ in the recording solutions and adding K^+^ channels inhibitors tetraethyl ammonium (TEA) and 4-aminopyridine (4-AP) in the bath solution. The isoosmotic solution contained the following chemicals (in mM): 80 choline-Cl, 20 TEA-Cl, 2.5 KCl, 1.25 NaH_2_PO_4_, 2 4-AP, 4 MgCl_2_, 26 NaHCO_3_, 11 glucose, 0.0005 TTX, and 50 mannitol (310±5 mOsm/Kg-H_2_O). The hypoosmotic solution was made by omitting mannitol (260±5 mOsm/Kg-H_2_O). All the solutions were bubbled with 5% CO_2_/95%O_2_, resulting in a pH = 7.3–7.4. The electrode solution contained (in mM) 120 NMDG, 120 gluconate, 4 MgCl_2_, 1 EGTA, 10 HEPES, 5 Na_2_-ATP and 0.3 Na-GTP (pH was adjusted to 7.25–7.27 at 20°C with NMDG, 296±5 mOsm). To calculate the active anion gradient in the recording solutions, the relative permeability of *P*_gluconate_/*P*_Cl_  = 0.12 [20] and *P*_HCO3_/*P*_Cl_ = 0.2 [21] have been taken into the consideration, which resulted in an [A^-^]_i_/[A^-^]_o_ = 22.4/115.7 (in mM) and an equilibrium potential for the activated anion conductance of *E*_A_ =  −41 mV. It should be noted that VRAC has a higher *P*_HCO3_/*P*_Cl_ = 0.64 [22], that yields a [A^-^]_i_/[A^-^]_o_ = 22.4/119.14 (in mM) and slightly negative *E*_A_ = −42 mV. Isoosmotic oxygen-and-glucose deprivation was achieved by substituting sucrose for D-glucose and bubbling solution with 95% N_2_/5% CO_2_ for at least 30 min. The same OGD solution was continuously bubbled with the same gases during slice recording in the chamber. As we reported before that in order to achieve an ischemic condition close to the *in vivo* state, the chamber perfusion was switched off 4 min after the normal aCSF being completely replaced by OGD solution [23].

### Electrophysiology

For *in situ* recording, individual slices were gently transferred into a recording chamber which was constantly perfused with isoosmotic solution (2.5 ml/min). Whole-cell membrane currents in voltage clamp mode and membrane potential in current clamp mode were amplified with a MultiClamp 700A amplifier, and the analog signals were sampled by a Digidata 1322A interface. The data acquisition was controlled by pCLAMP 9.0 software (Molecular Devices, Foster City, CA) installed on a Dell personal computer. Low resistance patch pipettes were fabricated from borosilicate capillaries (OD: 1.5 mm; Warner Instrument Corporation, Hamden, CT) using a Flaming/Brown Micropipette Puller (Model P-87, Sutter Instrument Co., Novato, CA). When filled with NMDG-gluconate based electrode solution (see below), the electrode resistance was 5–8 MΩ. For whole-cell recordings, only if the initial seal resistance reached more than 2 GΩ in the cell-attached mode, was the cell membrane ruptured to form a whole-cell recording configuration. Membrane potentials were read in the “I = 0” mode when the recordings were performed in voltage clamp mode. Membrane capacitance (*C*_M_) and electrode access resistances (*R*a) were measured by using the “membrane test” protocol built into the pCLAMP 9.2 (Molecular Devices). Consistent with the report by Inoue et al.,[11], [12], the *R*a value measured from NMDG-gluconate based electrode solution was higher than that of the KCl-based solution. However, only those recordings that had their *R*a values below 25 MΩ were used for continued chloride current measurement with *R*a being compensated for by at least 70%. The *R*a value was also monitored throughout the recording, and those recordings where *R*a varied more than 10% during an experiment were not used. Patch pipettes were filled with the solution mentioned in the preceding section. All the experiments were conducted at room temperature (22–24°C).

### TO-PRO-3 iodide staining and quantification of neuronal viability

We used TO-PRO-3 iodide staining (TO-PRO-3-I) to analyze OGD induced CA1 pyramidal neuronal death in the presence or absence of VRAC inhibitors. Incorporation of TO-PRO-3-I into the cellular DNA as an index of membrane damage has been widely used in cell death analysis [17], [18], [24]. Acute slices were prepared with the same procedure as for electrophysiological recording (n = 5, P21 male rats). After recovery in the normal aCSF for 1 hour and another 20 min in the isoosmotic bath, the slices were transferred to another incubation chamber for a 25 min OGD treatment. All the solutions stated here were identical to the ones used for electrophysiological recordings. The slices were returned to the isoosmotic bath and randomly divided into three groups: 1) control in isoosmotic bath (OGD); 2) in the same bath plus 100 µM NPPB (OGD+NPPB), or 3) 10 µM DCPIB (OGD+DCPIB). Different treatments were lasted identically for 60 min. The viability of pyramidal neurons was determined by incubating the slices with 0.5 µM TO-PRO-3-I (Molecular Probes; Eugene, OR) for 20 min. TO-PRO-3-I fluorescence density in pyramidal neuron layer was examined using a Carl Zeiss LSM510 META confocal microscope set at the 630 nm line of the HeNe laser. Emission was filtered through a long pass 650 nm filter. Images of the hippocampus CA1 region were obtained at 25 µm depth from the slice surface, with the same acquisition settings used for all conditions. For data quantification, the TO-PRO-3-I fluorescence above background in the CA1 pyramidal layer was determined using the “density slice” option of the National Institutes of Health Image J program [25]. All the procedures were done at room temperature.

### Western blot analysis

For immunoblot analysis of relative level of caspase-3 and caspase-9 expression, slices were treated and grouped as noted in the above section. In each group, five slices were collected after receiving different treatments noted in above section, washed and harvested in an ice-cold buffer containing 15 mM CHAPS, 1 mM EDTA, 20 mM Tris-HCl (pH 7.5), 10 µg/ml soybean trypsin inhibitor, 0.05% tween-20 and 10 µM phenylmethylsulfonyl fluoride. The slices were homogenized using a Pro homogenizer (Oxford, CT). The resulted lysates were collected in Laemmli buffer and subjected to 14% sodium dodecyl sulfate-polyacrylamide gel electrophoresis and the separated proteins were transferred to polyvinylidene difluoride membrane (Bio-Rad). The relative levels of the caspase-9, caspase-3 and GAPDH proteins were determined by immunoblot analysis using polyclonal antibodies directed against caspase-9 (H-83), Caspase-3 (H-277) and GAPDH (FL-335) (Santa Cruz, CA). The membranes were incubated with each of the primary antibodies (5 µg/ml) at 4°C over night, and the membranes were washed extensively with Tris-buffered saline with 0.1% Tween and reincubated with secondary, peroxidase-labeled antibodies (goat anti-rabbit IgG in1∶1000 dilution, Dako, Carpenteria, CA) for 2 hrs. Following washes, the ECL (Amersham Corp.) chemiluminescence detection system was applied to membrane to generate measurable signals. The resulting bands were analyzed densitometrically with a VersaDoc 5000 Imaging system (Bio-Rad Laboratories, Hercules, CA). The image intensities of caspas-9 and- 3 were normalized with GAPDH protein band density.

### Solutions and reagents

Tetraethyl ammonium (TEA), 4-aminopyridine (4-AP) and indanyloxyacetic acid (IAA-94) were purchased from Sigma (St. Louis, MO, USA). NBQX, AP-5, bicuculline, 5-nitro-2-(phenylpropylamino)-benzoate (NPPB), 4-(2-Butyl-6, 7-dichlor-2-cyclopentyl-indan-I-on-5-yl) oxybutyric acid (DCPIB), QX-314 were purchased from Tocris Bioscience (Ellisville, Missouri, USA). Other chemicals were purchased from Sigma (St. Louis, MO, USA). Stock solutions of NBQX, bicuculline and AP-5 were dissolved in water, and 0.1M IAA-94, 0.1M NPPB, 0.2 M furosemide 0.01 M tamoxifen and 0.2 M DNDS were dissolved in DMSO and stored in a −20°C freezer prior to use. These stock solutions were diluted to the final experimental concentration just before each experiment. The final concentrations used were: 300 µM IAA-94, 100 µM NPPB, 20 µM bicuculline, 40 µM AP-5, 30 µM NBQX, 400 µM DNDS, 200 µM furosemide, 10 µM tamoxifen and 10 µM DCPIB. In the bath solutions, the DMSO concentrations varied within 0.1% to 0.3%.

### Data analysis

Data are presented as means ± SEM. Student's *t*-test was performed to assess the statistical significance of the difference before and after treatment in the same experimental group. One-way ANOVA test was performed to determine the statistical significance of the differences between 2 experimental groups. Differences were considered significant at *p*<0.05 (indicated as *) or *p*<0.01(indicated as **).

## Results

### Functional expression of VRAC currents in rat hippocampal CA1 pyramidal neurons

**Figure 1:**
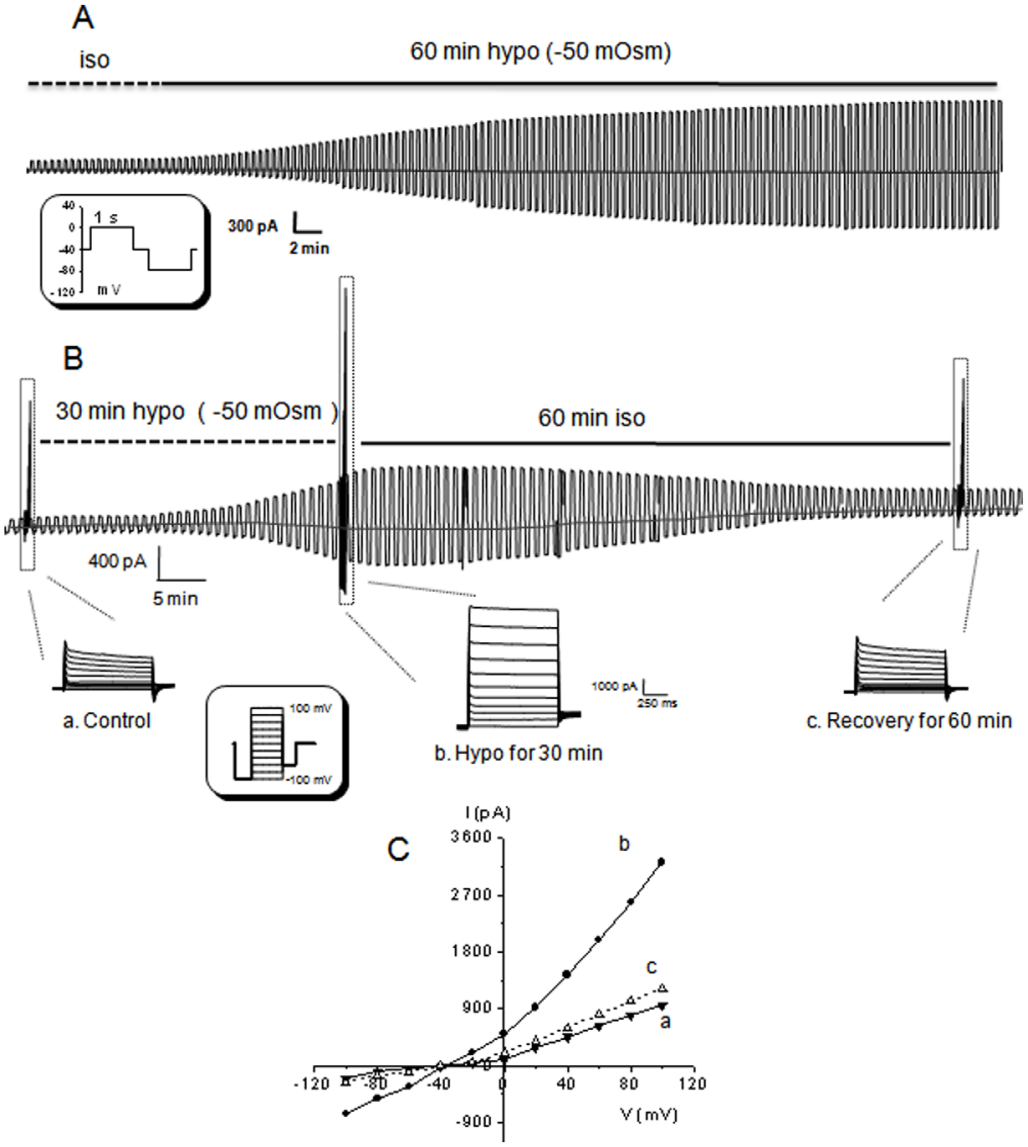
Expression of VRAC currents in CA1 pyramidal neurons in hippocampal slices. **A**, Shows a hypoosmotic medium -activated -chloride conductance (HAC) from a pyramidal neuron. After initial recording in the isoosmotic medium (iso, dashed line) as control, the perfusion was switched to the hypoosmotic medium (hypo, −50 mOsm) for 60 min. The neuronal Na^+^, Ca^2+^ and K^+^ channel conductances were pharmacologically inhibited (see Methods). The cell was held at −40 mV in the resting condition, and a pair of alternate voltage pulses at ±40 mV was delivered to the cell every 15 second. Each test pulse in the pair was 1 second long and was separated from each other by 300 ms at −40 mV resting voltage (see the shadowed inset in **A** for protocol). Because each series of paired alternate pulses was delivered every 15 s, the time scale bar shown under **A** includes all the unrecorded time periods, or the duration of alternate pulses induced currents are not proportional to the applied time scale. A progressive increase of chloride conductance was recorded over a 60 min of hypo exposure. **B**. A whole-cell chloride conductance recording with 30 min of hypo exposure. The HAC slowly inactivated after switching the perfusion to the iso. In the same recording, a voltage step protocol was delivered to the cell at the times indicated as “**a**”, “**b**” and “**c**” that represent the chloride currents at control, HAC and recovery, respectively. The I-V curves in **C** were at times of “a” and “b” and constructed by plotting the steady-state currents against the applied voltages, ranging from −100 mV to +100 mV in a 20 mV increments (see shadowed inset in **B**). In all the I-V curves in **C**, the chloride conductance was outwardly rectifying and reversed at around −40 mV.

Hippocampal pyramidal neurons were identified in the CA1 pyramidal neuron layer as we have recently described [17], [18]. To selectively study VRAC chloride currents, the voltage-gated Na^+^, Ca^2+^ and K^+^ channel currents were inhibited by combining ion substitution and use of channel inhibitors in the recording solutions (see Methods). When the neurons were held at −40 mV, a pair of alternate voltage pulses at ±40 mV induced only less than 250 pA residual whole-cell currents (Fig. 1A, B) and the currents were reversed at −37.5±0.7 mV (n = 11, Fig 1B-a, and C). This reversal potential was very close to the predicted *E*_A_ of −41mV (see Methods), therefore, the solutions were suitably designed for studying VRAC. It should be noted that the residual chloride whole-cell conductance was outwardly rectifying, indicating the expression a resting chloride conductance in rat CA1 pyramidal neurons, which is similar to the basal level activity of VRAC currents of barrel cortex neurons [12].

The alternate voltage pulses noted above were delivered every 15 seconds to the recorded neurons to monitor hypoosmotic medium (hypo) induced membrane currents (inset in Fig. 1A). Upon perfusion of slices with −50 mOsm hypo (see Methods), a hypoosmotic-activated-conductance (HAC) appeared progressively with time that took around 55 min to reach the steady-state level (Fig. 1A, n = 3). Shortening the hypo treatment time to 30 min, the HAC was readily reversible after switching the perfusion back to the isoosmotic solution (iso) (Fig. 1B). Outward rectification of whole-cell currents is a hallmark of VRAC that can be better characterized by a voltage step protocol reported before [12], [26]. The voltage step protocol induced a whole-cell conductance showing the same outwardly rectifying I-V relationship as recently reported from a study of mouse barrel cortex neurons in slices [12] (Fig. 1B, a–c). These results demonstrate that the HAC in pyramidal neurons is mediated predominantly by chloride selective anion currents that behaved with typical VRAC-like outward rectification (Fig. 1B-b, c). Time-dependent inactivation of VRAC currents induced by large depolarization voltage steps, i.e., +100 mV, has been shown in several cell types [3], [26]. In hippocampal pyramidal neurons, this feature was more pronounced in the anion currents recorded from basal, but less evident from hypo conditions (Fig.1B, a, b), which is consistent with the VRAC of barrel cortex neurons in brain slices [12].

### Pharmacological characteristics of hypoosmotic-activated-conductance (HAC) of pyramidal neurons

**Figure 2:**
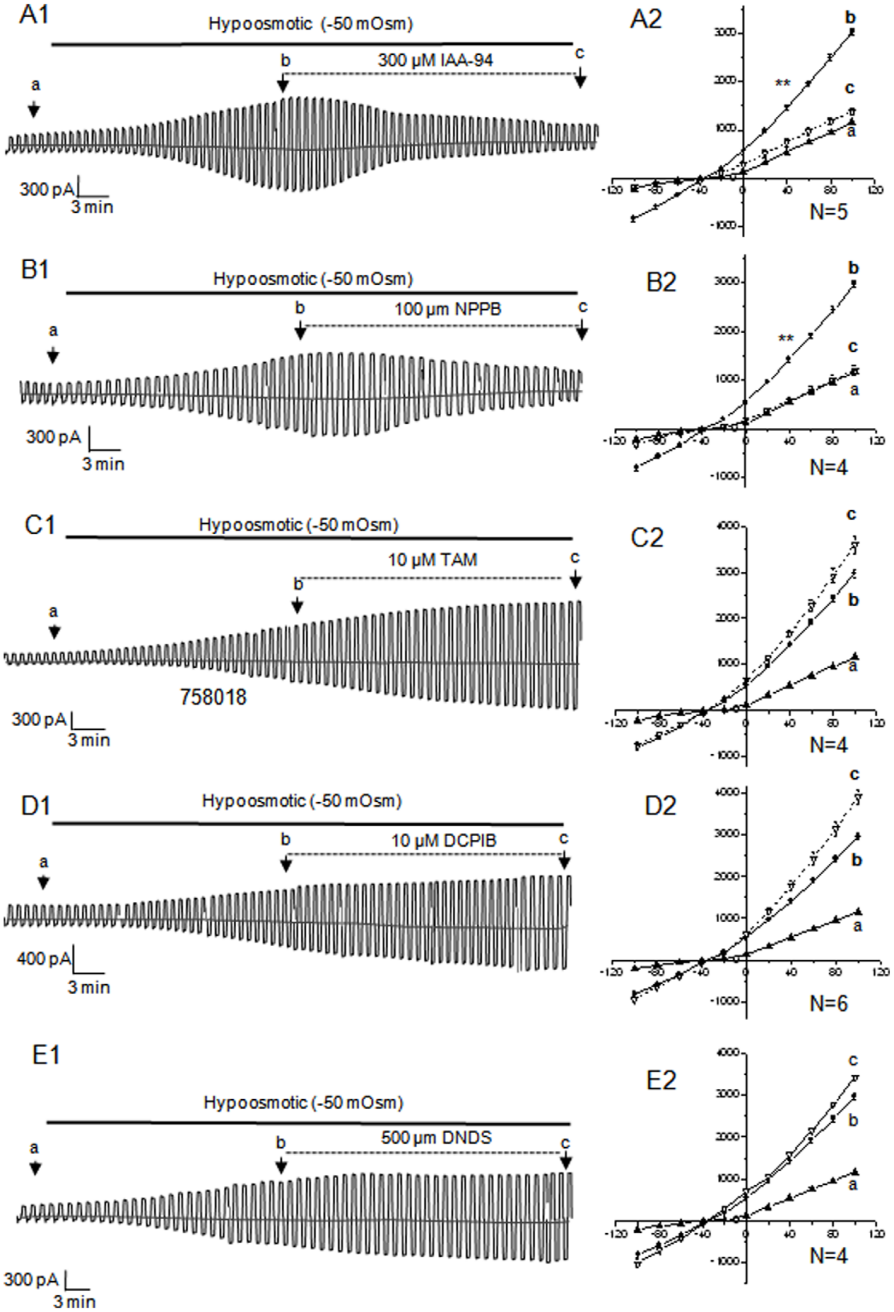
The pharmacological characteristics of neuronal HAC. The left panel recordings are HACs of 5 different pyramidal neurons. After 25 min hypo exposure and HAC induction, a given VRAC inhibitor was added in the perfusion solutions as indicated, and the drug treatments were lasted for 30 min. The anion currents were induced by the alternate voltage pulses (see description in Fig. **1A**). At the time points indicated as “**a** (control in iso)”, “**b** (25 min in hypo)” and “**c**” (30 min after addition of a putative VRAC inhibitor), the voltage step protocol described were delivered as in Fig. 1A and the resulting I-V curves are shown on the right hand panel next to the recording. According to the results presented in Fig. **1A**, the amplitude of HAC should increase over a total of 55 min in hypo medium. Thus, should a putative VRAC inhibitor be effective, the HAC would be inhibited. Otherwise, HAC would further increase in the following 30 min of hypo stimulation. Accordingly, 30 µM IAA-94 (**A1**) and 100 µM NPPB (**B1**) were identified as effective inhibitors, while 10 µM tamoxifen (TAM, **C1**), 10 µM DCPIB (**D1**) and 500 µM DNDS (**E1**) were ineffective. I-V curves represent the mean ±SEM (vertical bars) for 4–6 recordings. **. Indicates a statistical significance of difference at *p*≤0.01 in the +40 mV step induced current amplitudes between the time points of “**b**” and “**c**”. The difference in the current amplitudes induced by voltage steps more positive than +40 mV also reached the same *p* value.

VRAC currents can be inhibited by several pharmacological agents [3], [27], [28], however, the VRAC currents recorded from different cell types varied in their sensitivities to these inhibitors, e.g., the VRAC of the cultured cortical neurons were sensitive to IAA-94, NPPB, phloretin, SITS and DIDS, but not to tamoxifen [11], [29]. Because the HAC induced by a 25 min hypo could always be reversed to the control level after hypo withdrawal, we used 25 min hypo exposure to establish the pharmacological profile of HAC induced from CA1 pyramidal neurons. The effect of a given inhibitor was analyzed by comparing the difference of the voltage steps induced currents prior and at the end of drug treatment as indicated by the arrows in Fig. 2 (the steps induced current traces are not shown). The percentage inhibition of HAC by a given inhibitor was measured at the +100 mV step. All the I-V curves presented in Fig 2 were the averaged current amplitudes from a group of cells (n = 4–6). At +100 mV, the HAC were almost completely inhibited by 100 µM NPPB (97.6±5.0%, n = 4) and by 300 µM IAA-94 (88.5±5.0%, n = 5%) (Figs. 2, A1-2, B1-2). In contrast, 10 µM tamoxifen, 10 µM DCPIB and 400 µM DNDS increased the HAC by 30.2±11.0% (n = 4), 46.8±9.0% (n = 6) and 20.9±7.0% (n = 4), respectively (Fig. 2C1-2, D1-2, E1-2). This pharmacological profile is in good agreement with the VRAC reported from barrel cortex neurons in brain slices [12], [29]. Although test of type I Eisenman anion permeability sequence would provide additional evidence of VRAC involvement in HAC [30], however, the difficulty in achieving a full substitution of anions in the ambient of patched neurons with the use NaHCO_3_-based aCSF has limited us to pursue these data in slice recording.

### Neuronal VRAC activation in the post-OGD reperfusion phase

**Figure 3:**
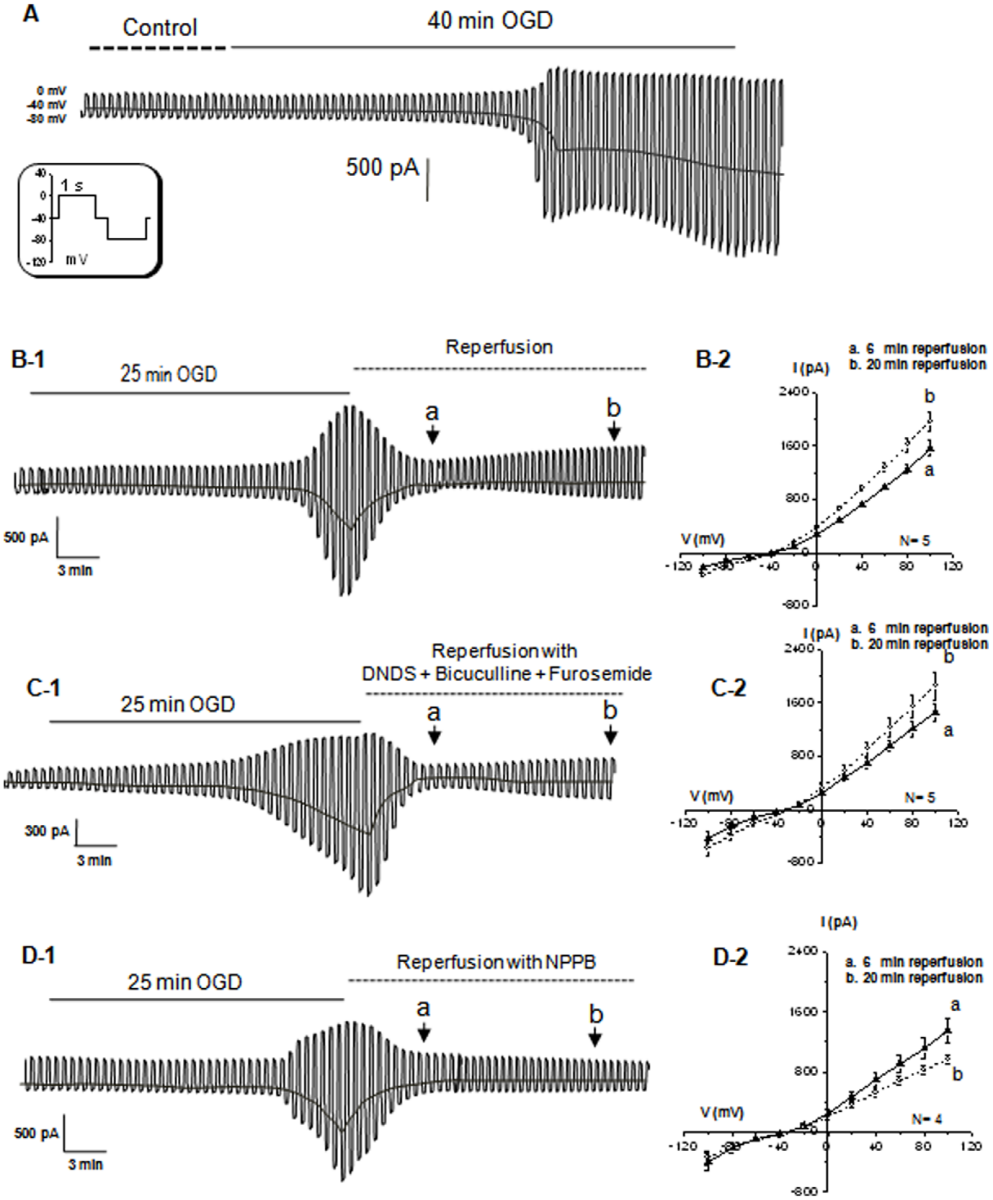
The post-OGD VRAC induced from rat hippocampal pyramidal neurons. **A**. Shows a neuronal recording during a 40 min of OGD perfusion. The OGD first induced a progressive activation of membrane conductance accompanied by a downward shift in the holding currents, and was then followed by an anoxic depolarization (AD) at 25 min after OGD onset. A 40 min OGD treatment typically resulted in an irreversible change in neuronal electrophysiology. For the recordings shown in **B**-1, **C**-1 and **D-**1, the OGD exposure was shortened to 25 min, where the OGD-induced neuronal electrophysiological changes were readily reversible at ∼6 min after withdrawal of OGD (reperfusion). In the reperfusion stage, the voltage step protocol was delivered at the time points of “**a**” and “**b**” to obtain the I-V curves (**B**2, **C**-2 and **D**-2, the voltage step induced current traces are not shown). The presence of a strong outwardly rectifying chloride conductance and a progressive increase of conductance in the reperfusion stage were disclosed by the I-V curves shown **B**-2, where the I-V curves obtained from 6 min and 20 min post-OGD can be compared. Both of the I-V curves showed a strong outward rectification and reversed at −40 mV. During the time period from 6 min to 20 min, the amplitude of the outward currents at +100 mV increased by 32% (1542±116 pA at “**a**” *vs*. 2041±65 at “**b**”). *: indicates a statistical significance of difference at *p<*0.05. In the recording of **C**-1, an inhibitor cocktail for Cl^-^ cotransporter and GABA_A_, i.e., 200 µM furosemide+400 µM DNDS+20 µM bicuculline, was applied to the reperfusion solution that did not prevent the outgrowing of outward chloride currents (**C**-2, 1460±126 pA at “**a**” *vs*. 1857±207 at “**b**”, *p*>0.05). The recording in **D**-1 showed that 100 µM NPPB not only prevented the outward anion conductance from further growing, but actually inhibited the outward currents to below the control level measured at 6 min in the reperfusion stage (1347±156 pA at “**a**” *vs*. 1013±89 at “**b**”).

We next used oxygen-and-glucose deprived (OGD) to simulate ischemic conditions and determine whether VRAC could be activated as a consequence of OGD treatment and the mechanism accounting for it. After perfusion of slices with OGD for 40 min, the neuronal recording first showed a progressive inward shift in the holding currents and increase in membrane conductance (Fig. 3A, n = 3). At 24.7±0.3 min after OGD onset (n = 11), a sudden downward shift in the holding currents and opening of a large membrane conductance was followed, which is a OGD induced neuronal electrophysiological change, termed anoxic depolarization (AD) and typically occurred ∼8 min after treatment of slices with the standard OGD solution [17], [18], [31]. Requiring a much longer OGD stimulation for AD induction should be largely due to the low Na^+^ and zero Ca^2+^ ions used in our bath solution, which diminished the influx of Na^+^ and Ca^2+^ through their respective voltage-gated channels and ionotropic glutamate receptors [17], [18], [32]. In view of a large downward shift in holding potential, corresponding to a positive shift of membrane potential (Fig. 3A), OGD induced hemichannels opening could also contribute to the AD [33]. Nevertheless, because 40 min OGD treatment always resulted in an irreversible damage to the recordings, we shortened the OGD to 25 min, where the AD could be readily induced but was still reversible (Fig 3B-1). Under this condition, the OGD-induced neuronal electrophysiological changes recovered to the control level within 5–6 min after OGD withdrawal (Fig. 3B-1). Thereafter, an outwardly rectifying current component progressively appeared during the following 20 min post-OGD reperfusion stage (Fig. 3B-1, b). Such a time-dependent increase of post-OGD outward conductance is more clearly shown in the I-V curves in Fig. 3B-2. Specifically, the amplitude of the outward currents was increased by 26% at the end of 20 min compared to the initial 6 min in the post-OGD stage (Fig. 3B1-2, 1542±116 pA at 6 min “a”, 1910±140 pA at 20 min “b”, +100 mV, n = 5). The currents were mostly carried by chloride anions but not hemichannels, because the whole-cell currents were reversed precisely at the *E*_A_ of −40 mV, and activation of latter should shift reversal potential to around zero potential [33].

Hippocampal pyramidal neurons also express ionotropic GABA_A_ receptors, HCO_3_^-^/Cl^-^ and electroneutral Na^+^-K^+^-2Cl^-^(NKCC)/K^+^-Cl^-^ (KCC) co-transporters [34], [35]. Although neither NKCC nor KCC activation generates measurable whole-cell currents, to test the contribution of GABA_A_ activation to the post-OGD anion currents, inhibitors for NKCC (400 µM DNDS) and KCC (200 µM furosemide) were also present to eliminate any cross membrane anion movement via NKCC and KCC. As shown in Fig. 3C-1-2, GABA_A_ inhibitor (20 µM bicuculline) did not prevent the outward anion currents from further increase, i.e., the amplitude of current increased by 27% at 20 min compared to at 6 min in the post-OGD stage (1461±127 pA at 6 min *vs*. 1857±208 pA at 20 min, n = 5) (Fig. 3C-1, 2). Therefore, activation of neuronal GABA_A_ receptor unlikely contributed to the post-ODG chloride conductance significantly.

**Figure 4:**
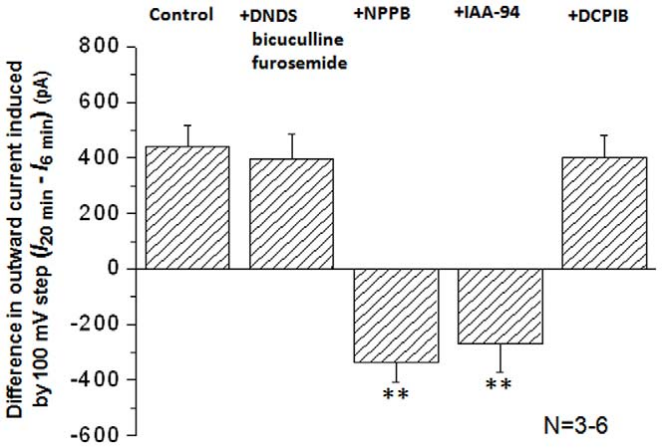
The pharmacology of post-OGD chloride conductance in neurons. The values in y-axis are the differences of the outward current amplitudes between 6 min and 20 min, *I*_20 min_ –*I*_6 min_, in the post-OGD stage. The outward currents were taken from the voltage step at +100 mV. The post-OGD outward chloride currents were not sensitive to the inhibitor cocktail containing DNDS, bicuculline and furosemide and DCPIB, but sensitive to 100 µM NPPB and 300 µM IAA-94. The latter two inhibitors attenuated the outward currents to the level below the current amplitudes measured at 6 min (445±75 pA in control, −334±72 pA in NPPB and −226±106 in IAA-94). ** Indicates a statistical significance of difference between the control and a tested inhibitor at *p*<0.01.

In the following experiment, we sought to determine the NPPB effect on the post-OGD VRAC currents. It has been shown that NPPB may have a minor effect on HCO_3_^-^/Cl^-^ co-transporter and NKCC/KCC activity. Therefore, we chose to add the inhibitors used above in the reperfusion solution. More importantly, this should allow isolation of an NPPB effect targeting specific to VRAC. In the presence of 100 µM NPPB in the reperfusion solution, not only were the post-OGD VRAC currents, but the basal level of post-OGD anion currents were inhibited by 25% (1348±157 pA at 6 min *vs*. 1014±90 pA at 20 min, n = 4) (Fig. 3D1-2, Fig. 4). Likewise, IAA-94 produced the same inhibitory effect on the post-OGD VRAC (not shown). These results demonstrated the presence of active VRAC currents early on, which develop further in the post-OGD reperfusion stage.

In line with what we've shown earlier that neuronal HAC was insensitive to the VRAC inhibitor DCPIB, the post-OGD VRAC currents also could not be inhibited by 10 µM DCPIB. The pharmacological profile of the post-OGD VRAC currents is shown in Fig. 4, where the sensitivity of anion currents to a given inhibitor is determined by the difference in the current amplitude that were measured at 6 min and 20 min post-OGD (*I*_20 min_ –*I*_6 min_), respectively. The currents used for calculation were induced by +100 mV voltage step.

### Mechanisms underlying OGD induced VRAC activation

**Figure 5:**
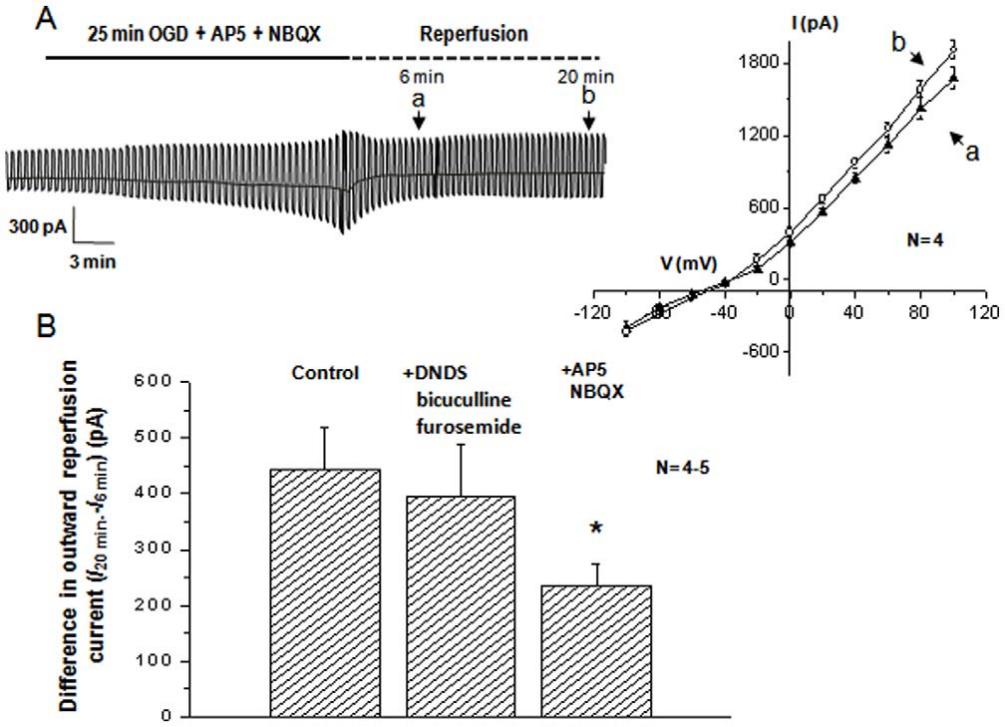
OGD induced VRAC activation requires Na- +loading through glutamate receptors. **A**. In the presence of 40 µM AP-5 and 30 µM NBQX in the OGD solution to inhibit ionotropic glutamate receptors, the 25 min OGD induced neuronal electrophysiological changes seen in the Fig. 3A were largely attenuated. Also, the activation of VRAC in the reperfusion stage was significantly inhibited. **B**. Shows the differences in the outward current amplitudes between 6 min and 20 min post-OGD under the following conditions: 1) control; 2) in the presence of DNDS+ bicuculline +furosemide in the OGD and 3) in the presence of AP-5+NBQX in the OGD solution. * Indicates that the difference between the control and AP-5+NBQX groups was statistically significant at *p*<0.05.

Excessive Na^+^-loading through ionotropic glutamate receptors has been showed to initiate an impaired regulatory volume regulation that eventually lead to the necrotic cell death [12], [36]. We next asked whether OGD-induced glutamate receptor activation is required for the neuronal VRAC activation in the reperfusion stage. To address this question, neuronal NMDA inhibitor, 40 µM AP-5, and 30 µM of AMPA receptor inhibitors NBQX, were added together in the OGD solution. We found that not only was the OGD activated membrane currents decreased, but the AD that always occurred at the end of 25 min OGD treatment (Fig. 3), was also prevented. Noticeably, in the presence of AP-5 and NBQX in the OGD solution, the post-OGD anion conductance was significantly reduced by 53%. Specifically, the values of *I*_20 min_ –*I*_6 min_ were 444.5±75.1 pA in the OGD control group and 235.3±38.1 pA in the OGD+AP-5+NBQX group (n = 4 for each group, *p*<0.01,) (Fig. 5A–B). In contrast, addition of a cocktail containing DNDS/bicuculline/furosemide in OGD solution did not significantly affect the post-OGD VRAC currents (396.1±92.4 pA, n = 5, *p*>0.05, Fig. 5B). Student's *t* test was used for above data analyses. These results demonstrate that OGD induced stimulation of ionotropic glutamate receptors plays a crucial role in mediating neuronal VRAC activation.

It should be noted that Allen et al. [37] have shown that OGD induced early exocytotic release of GABA lead to neuronal swelling and contribute to the OGD-induced VRAC activation. However, bicuculline failed to significantly inhibit post-OGD VRAC currents. Different chloride gradients and holding potentials used between the two experiments should be primarily attributable to this discrepancy.

### VRAC activation during the post-OGD period contributes to neuronal damage

**Figure 6:**
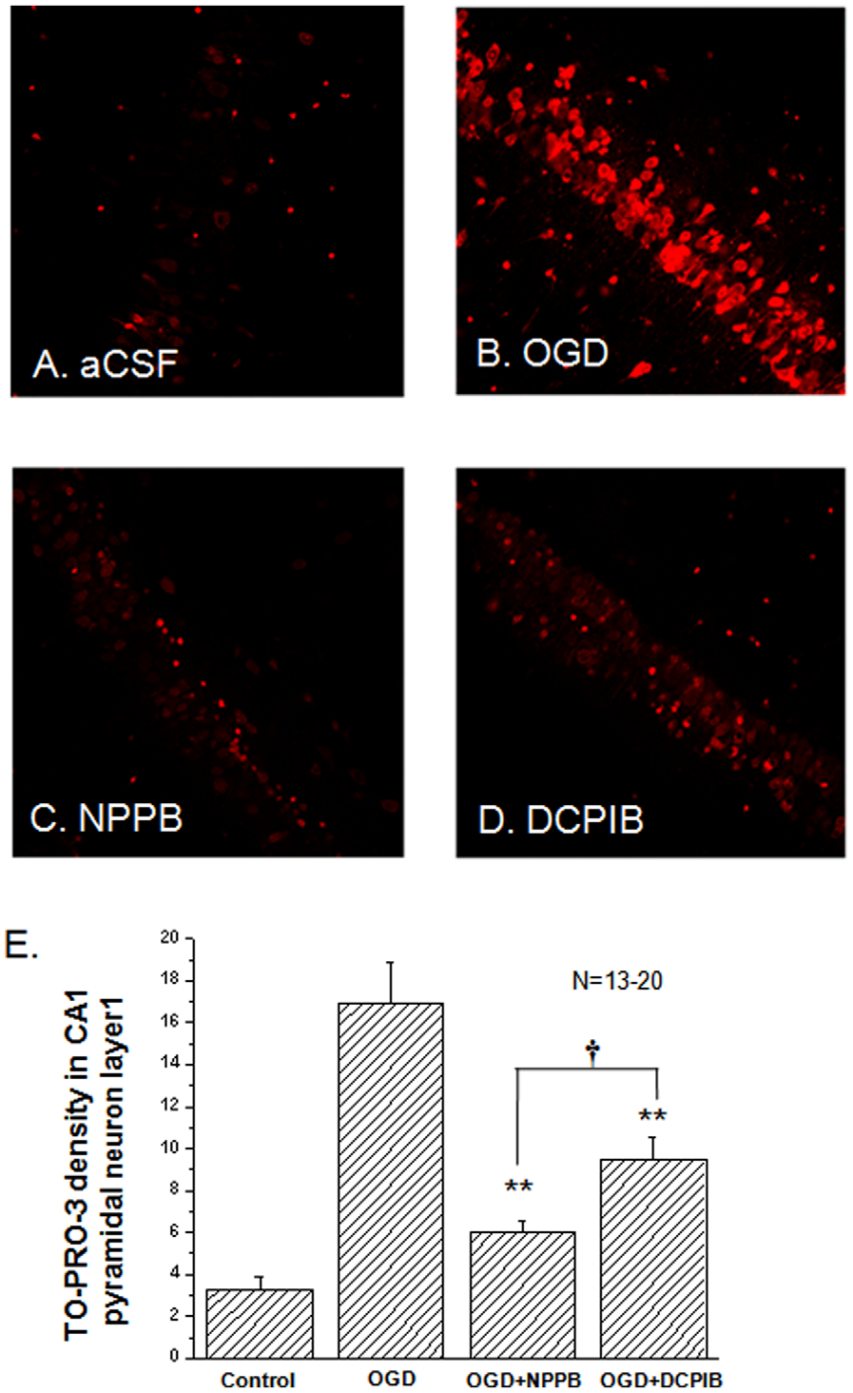
The effects of NPPB and DCPIB on OGD induced pyramidal neuron death. Besides the control group (in aCSF, **A**), the hippocampal slices were randomly divided into three groups after 25 min OGD treatment to receive the following post-OGD treatment: 1) in bath solution (**B**. OGD); 2) bath solution+100 µM NPPB (**C.** NPPB); and 3) bath solution+10 µM DCPIB (**D.** DCPIB). The fluorescence density of the TO-PRO-3-I staining is proportional to the neuronal death. Between the aCSF control and the OGD group, the neuronal death increased by 5.3 fold (3.2±0.6 in aCSF *vs*. 16.9±1.9 in OGD, n = 13). The neuronal death was reduced to 6.0 ±0.5 (n = 20) by 100 µM NPPB, and to 9.4±1.1 (n = 20) by 10 µM DCPIB. Both NPPB and DCPIB were added in the reperfusion bath solution to inhibit post-OGD VRAC. All the fluorescence intensity values are arbitrary. **. The difference between the OGD and NPPB or DCPIB groups is statistically significant at *p*≤0.01t. **†**. The difference between the NPPB and DCPIB groups is statistically significant at *p*≤0.05.

The impact of OGD-induced VRAC activation on neuronal death was determined by TO-PRO-3-I staining of CA1 pyramidal neurons in the presence and absence of NPPB in the OGD solution as NPPB shown the strongest inhibition on post-OGD VRAC currents. Although DCPIB did not inhibit neuronal VRAC in the present study, a potent inhibition of DCPIB on VRAC currents of cultured astrocytes promoted us to include this inhibitor in this study as a control. In the presence of NPPB or DCPIB, the TO-PRO-3-I fluorescence density was reduced from 16.9±1.9 (n = 13) to 6.0±0.5 (n = 15) and 9.4±1.1 (n = 19), respectively (*p*<0.01, Fig. 5A–C). Thus the neuroprotection produced by NPPB was more pronounced compared to DCPIB (*p*<0.05, Fig. 6D). Nevertheless, the fact that DCPIB was also able to produced a sizable neuroprotection implies a different VRAC target of DCPIB action, such as inhibition of astrocyte VRAC [26]. However, the inhibition of neuronal VRAC by NPPB does contribute significantly to the overall neuroprotection under cerebral ischemia conditions.

**Figure 7:**
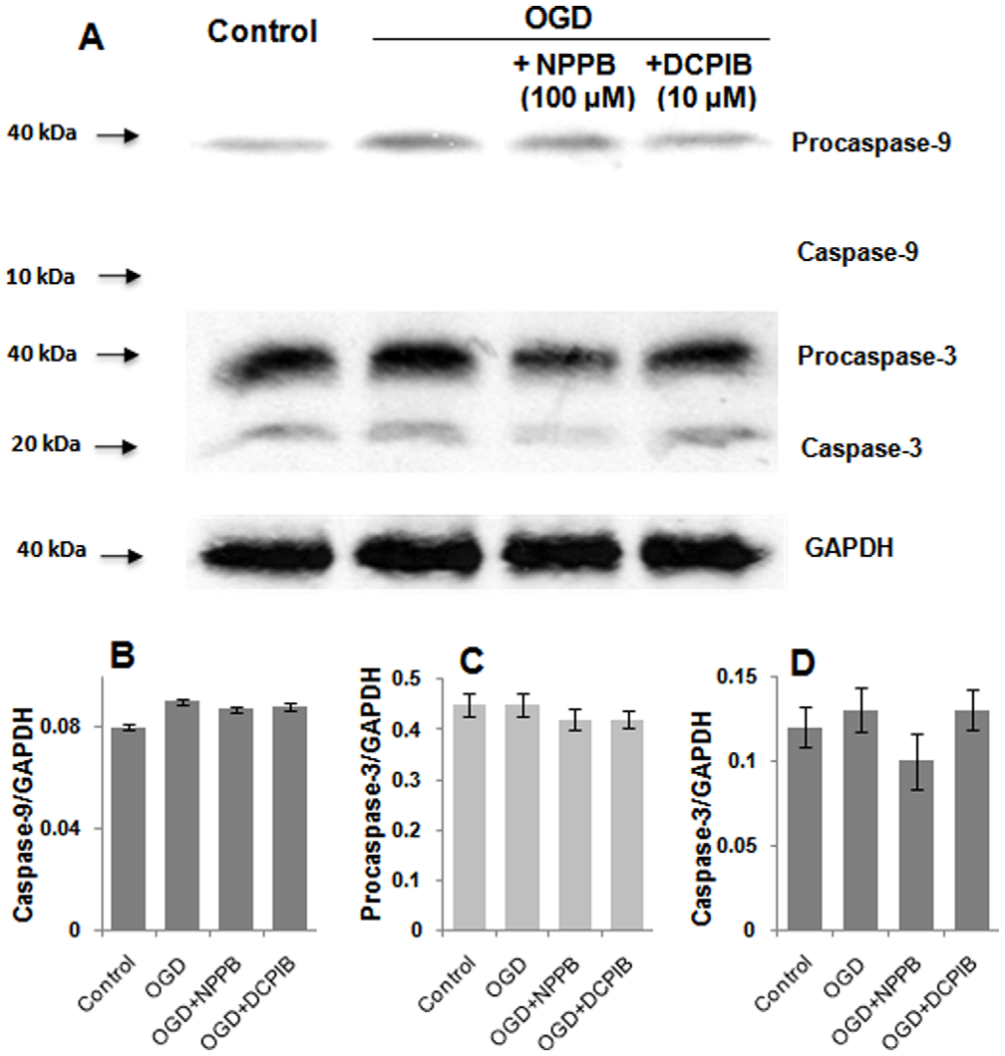
Short-term OGD did not change the levels of caspase-9 and -3. **A**. Western blot analysis was used to detect caspase-9, caspase-3 and their precursors from lysates of slices pretreated with different conditions as indicated. 100 µg whole cell proteins in each lane were used in western analysis using anti- caspase-9, caspase-3 and GAPDH. **B-D**. Bar graphs show the expression levels of procaspase-9, procaspase-3 and caspase-3 that are normalized to GAPDH (n = 3). The difference was not statistically significant among different treatment groups in each category shown in **B**–**D**.

It was possible that apoptotic neuronal death a result of short-term OGD treatment may also account for the observed neuronal death. OGD induced mitochondria release of cytochrome c that in turn sequentially recruit and activate caspase-9 and caspase-3, to induce apoptotic chromatin condensation and DNA fragmentation. To further determine if apoptotic neuronal death was causative to OGD induced acute neuronal death, we analyzed the expression levels of program cell death proteins caspase-9 and -3 in the experimental groups described above, i.e., control (aCSF), OGD, OGD+NPPB and OGD+DCPIB. However, both pro-caspase-9 and active form of caspase-9 showed a similar expression level in control as well as other treatment groups, same was a low and even levels of pro-casepase-3 and caspase-3 among all the groups (Fig. 7). These results indicate that a 30 min OGD treatment could not induce significant apoptotic neuronal death.

## Discussion

Although the molecular identity remains unknown, the pathological involvement of VRAC in brain edema and ischemia has been extensively studied [1], [3], [30]. At the cellular level, however, our understanding of VRAC in the CNS cells has been mostly obtained from study of primary cell cultures [9], [11], [14], [26], [38], [39], that it remains to be determined whether neurons and astrocytes in the intact brain express VRAC similar to their counterparts in the cell culture conditions. In a recent *in vivo* study, release of excitatory amino acids from VRAC activated by focal hypoosmotic challenge has been characterized by two distinct modes [40]. In animal stroke model studies, VRAC inhibitors, such as NPPB, tamoxifen and DCPIB, were potent neuroprotectants [27], [28], [41], [42], [43], [44], but little is known in regard to their specific cellular targets. The present study demonstrates, for the first time, the functional expression of VRAC in the rat hippocampal pyramidal neurons in slices with a pharmacological profile closely resembling barrel cortex neurons also in slices [12]. In addition, we have shown that OGD activated neuronal VRAC are likely detrimental to the neurons in OGD-treated slices.

### Identification of VRAC in hippocampal pyramidal neurons

Without knowing the molecular basis, we based our identification of VRAC on the physiological criteria described from various other cell types [4], [45], [46], [47]. These criteria are: 1) activation of chloride currents by hypoosmotic challenge with a characteristic outwardly rectifying current profile, and 2) sensitivity of hypoosmotic induced currents to VRAC inhibitors NPPB and IAA-94. Although a time-dependent inactivation appeared when the VRAC currents were induced by large depolarization voltage steps in other cell types, a strong outward current inactivation was not always observed in neurons recorded from slices [12]. In the present study, the VRAC of hippocampal pyramidal neurons in slices appear to share the same characteristic with barrel cortex neurons. In addition, we have shown that CA1 pyramidal neurons also share a comparable VRAC pharmacology with barrel cortex neurons in culture and in brain slices; the VRAC currents were sensitive to NPPB and IAA-94, but not to tamoxifen (Inoue et al, 2005; Inoue et al., 2007).

Interestingly, the VRAC of CA1 pyramidal neurons were also insensitive to DCPIB, which differs markedly from the VRAC currents of cultured astrocytes [26]. Based on this, one could speculate that the channels underlying the VRAC may differ between neurons and astrocytes. It should be noted that we used a relatively low 10 µM DCPIB (IC_50_  = 4.1 µM) [48] in this study, thus, we could not fully rule out a possible partial inhibition of neuronal VRAC by high dose of DCPIB. Also, lack of specific inhibitors for massively expressed astrocyte background K^+^ conductance makes a direct test of VRAC inhibitors on astrocyte VRAC in slices technically unfeasible [49], [50].

### OGD induced VRAC activation requires neuronal glutamate receptor mediated excitotoxic Na^+^ loading

The present study demonstrates, for the first time, that the activation of neuronal VRAC can be a consequence of OGD treatment in brain slices. The OGD induced VRAC showed the same electrophysiological and pharmacological characteristics as the VRAC conductance induced by hypoosmotic stimulation. However, the OGD induced VRAC becomes more evident in the post-OGD stage and progressively increase with time.

Cerebral ischemia triggers an early pathological activation of ionotropic glutamate and GABA receptors [13], [31], [32], [51]. The Na^+^ and Cl^-^ loading through this excitotoxic stimulation are associated with obligatory water influx that lead to cell swelling [5], [36], [52]. Should ischemic stimuli last only for a short time period, VRAC activation in swollen neurons could lead to Cl^-^ efflux that in turn restores the cell volume via regulatory volume decrease (RVD). However, excessive and prolonged Na^+^ and Cl^-^ loading and cell swelling could lead to an impaired RVD and ultimately to necrotic cell death [5], [36], [52]. In the present study, the requirement of excitotoxic Na^+^ loading for neuronal VRAC activation has been supported by the following two experiments. First, by allowing enough OGD exposure time for AD induction (Fig. 3) associated with a massive ionotropic glutamate receptor activation [32], we were able to record a progressively activated anion conductance with VRAC characteristics in the post-OGD stage. Second, by inhibiting the stimulation of ionotropic glutamate receptors (Fig. 5), we observed a significant reduction of post-OGD VRAC currents.

### Does excessive VRAC activation contribute to OGD induced neuronal death?

In the present study, we've shown that OGD-induced neuronal death could be significantly reduced by adding VRAC inhibitor NPPB in the reperfusion solution, therefore, VRAC activation appears to be detrimental to neurons suffering from OGD insults (Fig. 6). In view of the dependence of excitotoxic stimulation for neuronal VRAC activation, an impaired RVD mediated necrotic cell death should be responsible for the CA1 pyramidal neuronal death as being shown by TO-PRO-3-I staining. This notion was supported by an insignificant induction of apoptotic cell death proteins, caspase-9 and -3 (Fig. 7). In this study, we could only infer a detrimental role of VRAC form the facts that NPPB potently attenuated OGD induced VRAC currents as well as neuronal death, to what extent the VRAC activation reaches a threshold to trigger neuronal death remains to be determined.

Interestingly, although DCPIB did not inhibit neuronal VRAC currents, the inhibitor did produce a remarkable neuroprotection. Because DCPIB and NPPB inhibited VRAC of cultured astrocytes with a nearly equal potency [26], [48], a plausible explanation of DCPIB action would be due to its action on astrocytic VRAC. Accordingly, a stronger NPPB neuroprotection could be explained on a hypothetical basis of dual action of NPPB on OGD-induced neuronal as well as astrocytic VRAC.
